# Supplementary material for: Comparison of NADAL COVID IgG/IgM rapid test and DiaSorin Liaison SARS-CoV-2 S1/S2 IgG assay across different blood sources and substrates
Source: Microbiol Spectr. 2025 Jun 2;13(7):e03350-24. doi: 10.1128/spectrum.03350-24 (PMC12211023; doi:10.1128/spectrum.03350-24)
Supplement: Supplemental Figures — Fig. S1 to S3. [file spectrum.03350-24-s0001.docx]

**Comparison of NADAL COVID IgG/IgM rapid test and DiaSorin Liaison SARS-CoV-2 S1/S2 IgG assay across different blood sources and substrates.**

Supplemental document

**Figure S1: Interpretation of NADAL^®^ COVID-19 IgG/IgM serology kit results**


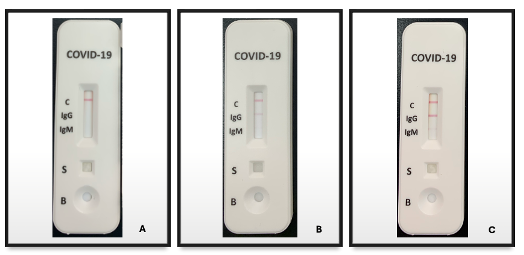


Panel A: Negative result indicated by a single line at the control (C) region, with no lines at the IgG or IgM regions. Panel B: Positive result for IgG antibodies, shown by lines at both the IgG and control (C) regions. Panel C: Positive result for both IgG and IgM antibodies, indicated by lines at the IgG, IgM, and control (C) regions.

**Figure S2: Instructional brochure on using the NADAL^®^ COVID-19 IgG/IgM provided to study participants.**


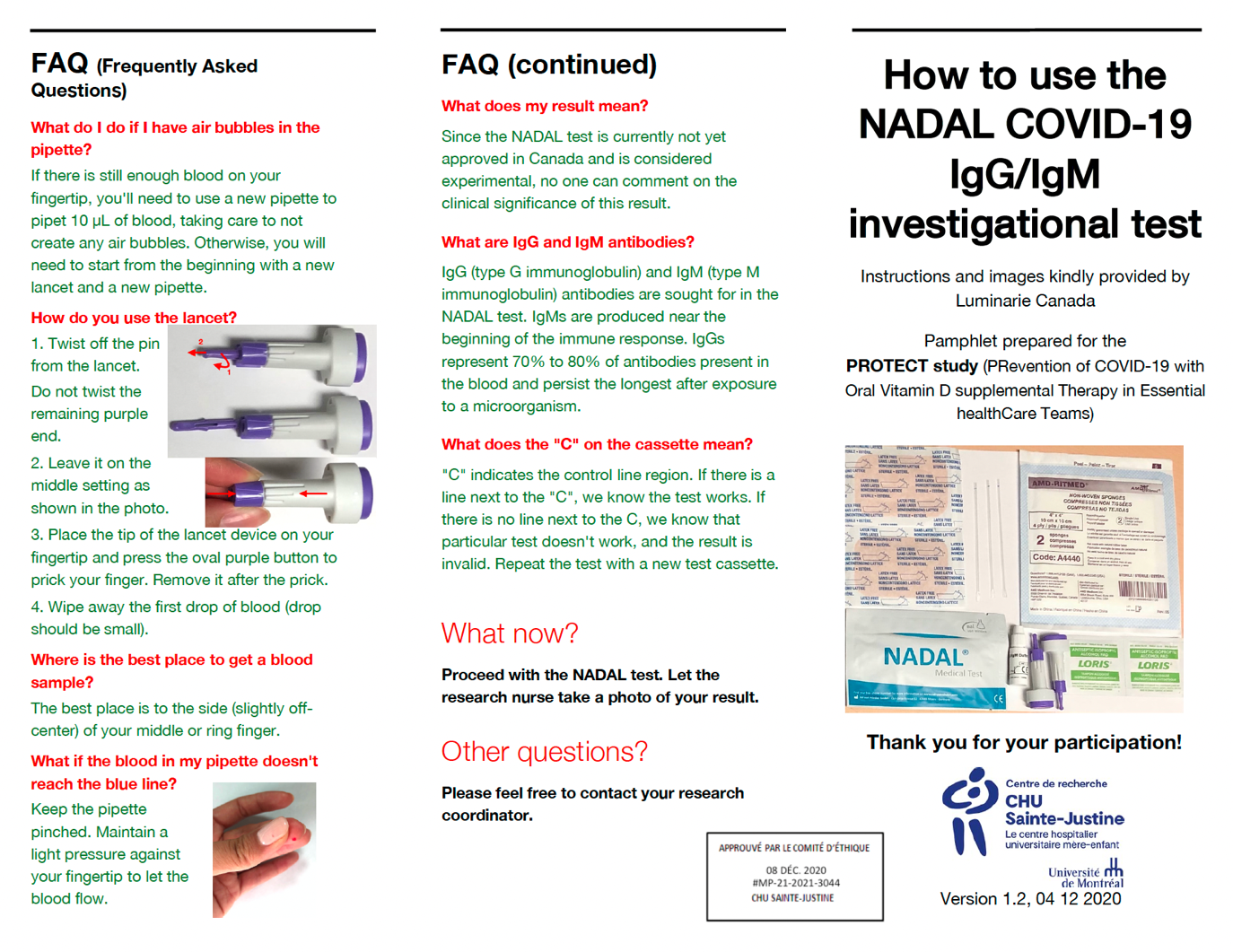


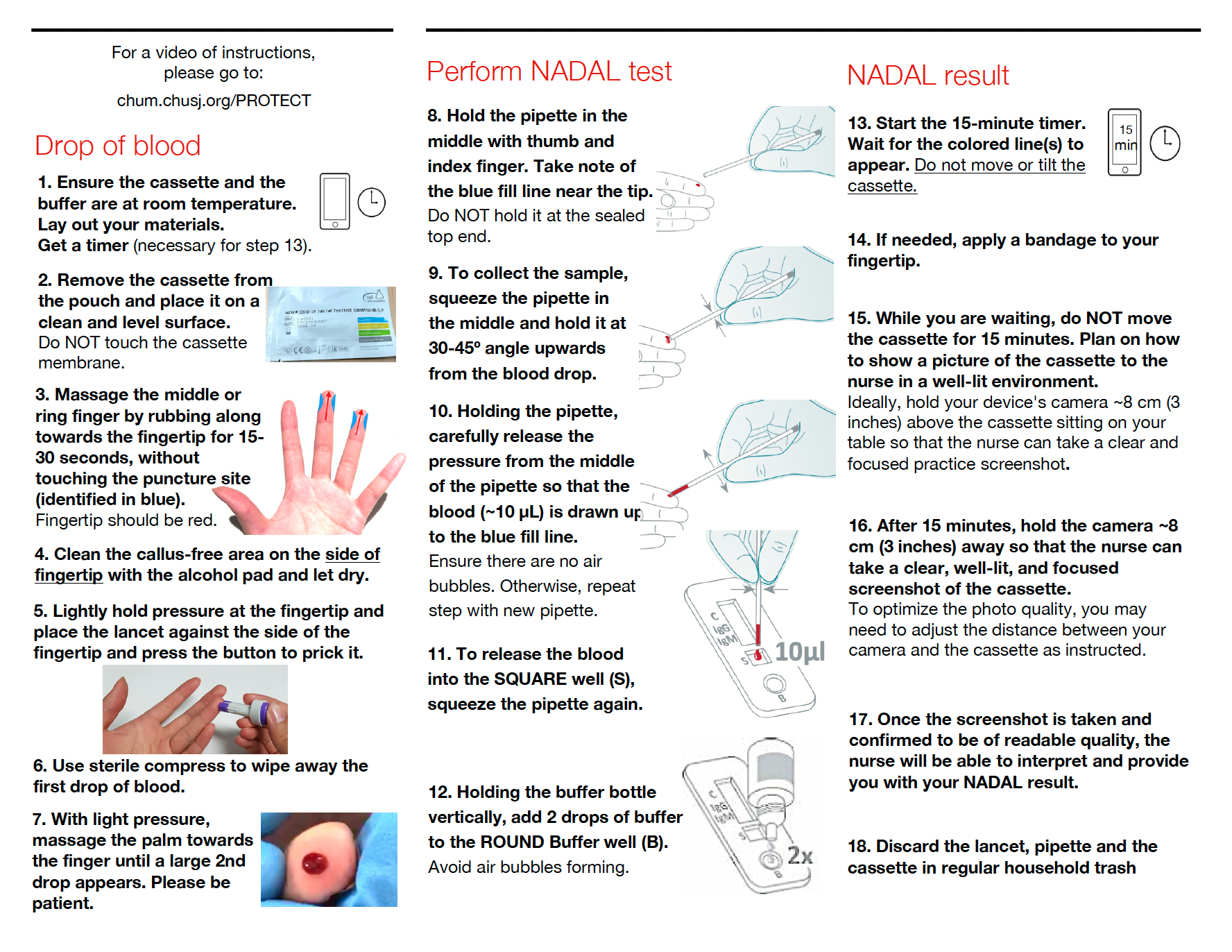
This figure presents the instructional brochure provided to study participants on using the NADAL^®^ COVID-19 IgG/IgM rapid test. The brochure contains step-by-step instructions and illustrations to ensure correct usage and interpretation of the test results by participants.

**
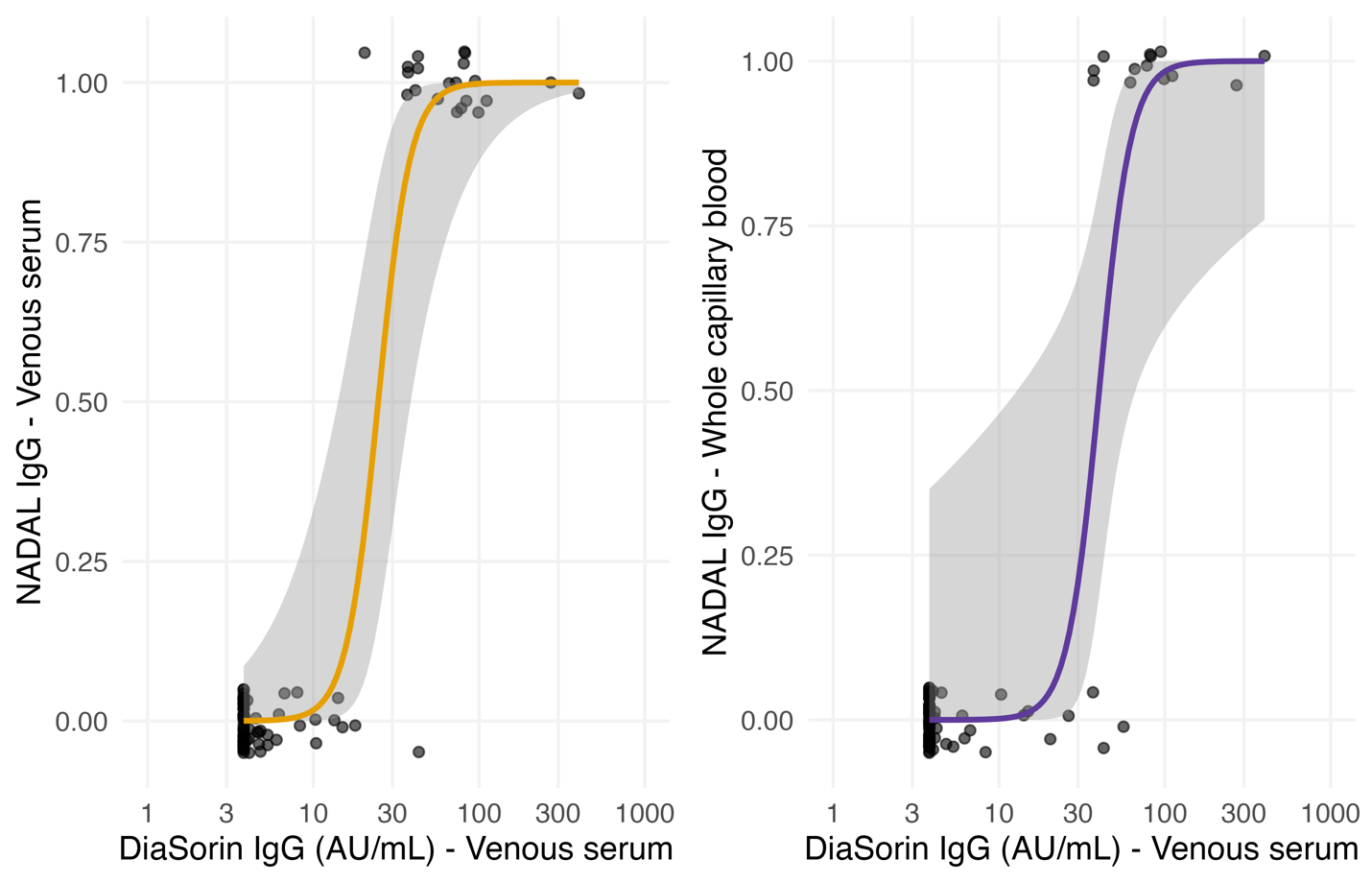
Figure S3: Association between DiaSorin IgG levels and NADAL IgG test results**

Scatter plots with logistic regression curves illustrating the relationship between DiaSorin IgG concentrations in venous serum (x-axis, log-transformed) and binary NADAL IgG test outcomes (y-axis). The left panel displays NADAL IgG results on venous serum, while the right panel shows NADAL IgG on whole capillary blood. Each dot represents an individual sample. The shaded grey areas indicate the 95% confidence intervals of the fitted logistic curves, capturing the transition from NADAL-negative to NADAL-positive results across the IgG spectrum.
